# Compact Interrogation System of Fiber Bragg Grating Sensors Based on Multiheterodyne Dispersion Interferometry for Dynamic Strain Measurements

**DOI:** 10.3390/s22093561

**Published:** 2022-05-07

**Authors:** Dragos A. Poiana, Julio E. Posada-Roman, Jose A. Garcia-Souto

**Affiliations:** Sensors and Instrumentation Techniques Research Group, Electronics Technology Department, University Carlos III of Madrid, 28911 Leganes, Spain; jposada@ing.uc3m.es (J.E.P.-R.); jsouto@ing.uc3m.es (J.A.G.-S.)

**Keywords:** nano-strain, vibrations, ultrasounds, dual optical frequency comb, phase-generated carrier, PGC, fiber Bragg grating sensors, dispersion interferometer, multiheterodyne sources, dual-drive Mach–Zehnder modulator

## Abstract

Dual-comb multiheterodyne spectroscopy is a well-established technology for the highly sensitive real-time detection and measurement of the optical spectra of samples, including gases and fiber sensors. However, a common drawback of dual-comb spectroscopy is the need for a broadband amplitude-resolved absorption or reflection measurement, which increases the complexity of the dual comb and requires the precise calibration of the optical detection. In the present study, we present an alternative dispersion-based approach applied to fiber Bragg grating sensors in which the dual comb is compacted by a single dual-drive-unit optical modulator, and the fiber sensor is part of a dispersion interferometer. The incident dual comb samples a few points in the spectrum that are sensitive to Bragg wavelength changes through the optical phase. The spectra reading is improved due to the external interferometer and is desensitized to changes in the amplitude of the comb tones. The narrow-band detection of the fiber sensor dispersion changes that we demonstrate enables the compact, cost-effective, high-resolution multiheterodyne interrogation of high-throughput interferometric fiber sensors. These characteristics open its application both to the detection of fast phenomena, such as ultrasound, and to the precise measurement at high speed of chemical-/biological-sensing samples. The results with a low-reflectivity fiber Bragg grating show the detection of dynamic strain in the range of 215 nε with a 30 dB signal to noise ratio and up to 130 kHz (ultrasonic range).

## 1. Introduction

A dual optical frequency comb (DOFC) [1,2,3] is a useful measurement tool that permits us to perform spectroscopy techniques [4] to study a certain interval of the spectra simultaneously [5,6,7,8,9]. It behaves as a broad source that is composed of optical discrete tones that are coherent and, at the same time, they unambiguously map onto a lower-frequency electrical domain where they are more easily detected. 

Fiber Bragg grating (FBG) sensors are in-fiber diffractive-pattern devices that can be used to sense physical magnitudes [10,11,12]. They are used for temperature and strain measurements since they act as an optical filter whose central wavelength, or Bragg wavelength, depends on the strain and temperature variation to which the sensor is applied. Typical sensitivities are about 1 pm/µε and 10 pm/°C, respectively [10].

The simplest approach to interrogate an FBG sensor relies on measuring the reflection spectrum. A broadband source is filtered with an FBG sensor and the center wavelength of the reflection is tracked with wavelength-sensitive detection, and therefore temperature and strain can be recovered. This can be conducted with an optical spectrum analyzer, and it can achieve a resolution of 1 pm of the optical wavelength, that is equivalent to 1 µε in mechanical displacement.

Another approach is to use a tunable laser. In this case, the wavelength of the laser is swept along a known interval of the optical spectrum, and therefore the amplitude of the light is modulated at the particular wavelengths when filtered by the FBG sensor. The result is a wavelength to time mapping and its analysis allows us to obtain the position of the FBG wavelength and its variation. Those systems have a resolution dependent on the sweeping parameters and usually achieve a sub-µε level.

An alternative to measuring the reflection is to use the dispersion profile [13]. This reading technique allows an increase in the overall sensitivity of the system compared to the typical reflection amplitude readout. This approach was proposed with a white light source and implies a low-coherence interfering process between a reference arm and the reflections from the FBG, as its structure is penetrated by different wavelengths [14].

In this paper, we propose to use a DOFC as the source of a dispersion interferometer to read the FBG changes through the optical phase. The employed DOFC architecture is functionally equivalent to an electro-optic DOFC that includes an acousto-optic frequency shift for disambiguating the detected tones [15]. However, instead of the acousto-optic modulator, which is more difficult to integrate in a compact set-up, our system uses an electro-optic in-chip architecture with a phase-generated carrier (PGC) technique [16,17,18]. We discuss the multiple carrier characteristic of this kind of modulation.

The proposed technique generates an unambiguous mapping of the dual-comb sidebands while, at the same time, it is implementable with a two-input, dual-drive Mach–Zehnder modulator (DD-MZM) [19]. We present the differences between the operating principles of the PGC modulation and the acousto-optic-based frequency shift in a DOFC. We also analyze its output amplitude in a steady state and conclude the superior mode-amplitude stability of the in-chip implementation over the discrete implementation.

Finally, we demonstrate the applicability of this system to detect surface acoustic waves and vibrations applied to an FBG sensor by means of a piezo-electric actuator (PZT). Previously, we proposed a DOFC fast readout of the FBG changes based on the reflected amplitude [20], where we obtained measurements of the dynamic strain of 0.32 µε (50 nε resolution) at 120 kHz. Here, we obtain an improvement of the sensitivity by using just two modes to detect the optical phase changes and a differential lock-in technique to improve the minimum detectable to the nε (nano strain) level, at mechanical vibration frequencies of up to 130 kHz.

The proposed system is calibrated with a reference instrument based on an auxiliary heterodyne interferometer. We use the attenuation of the sidebands of the heterodyne interferometer to obtain an absolute value of the strain amplitudes measured. Considerations on the general performance and stability of the source are also discussed in comparison to the discrete implementation.

## 2. Principle of Measurement

Strain is a primary magnitude of mechanical sensing. It reveals displacement or elongation (1) that can change dynamically as a result of the transduction of vibrations, acoustic emission or ultrasounds.

(1) ε=ΔlL0,

where Δl is the variation of the length experienced by the understudy element and $L_{0}$ is its initial length.

A practical optical gage to measure strain is an FBG sensor, in which the reflected wavelength changes with the strain (2) with a sensitivity of about 1 pm/µε.

(2) ΔλBλB=KB·ε=KB·ΔlL0,

where $\lambda_{B}$ is the Bragg wavelength (center of the reflected spectrum), $\Delta \lambda_{B}$ is the change of the Bragg wavelength with the strain and $K_{B}$ is the gage factor that considers the strain-optic coefficient. Considering a practical gage factor of 0.78, the sensitivity is 1.21 pm/µε for the 1550 nm wavelength and 1.03 pm/µε for the 1310 nm wavelength. The better performance of measuring the wavelength change (pm), the better the measurement of the strain (µε) with this sensor. For example, an optical spectrum analyzer with a 20 pm resolution allows the direct detection of 20 µε.

### 2.1. Electro-Optic Dual Optical Frequency Comb

The dual optical frequency comb is a high-performance architecture for optical spectra interrogation, and therefore it is applicable to read FBG. The working principle consists of generating two optical frequency combs at different frequency rates. Both combs come from the same optical source and are mutually coherent. We can merge them to obtain a multiheterodyne interference signal on a photodetector. The resulting electrical signal is a set of equally frequency spaced tones, each one corresponding to the beat of two optical tones. This method allows the independent recovery of both the amplitude beat and phase of the optical tones. A typical architecture implies an electro-optic generation of sidebands and an acousto-optic frequency shift [15].

**Figure 1:**
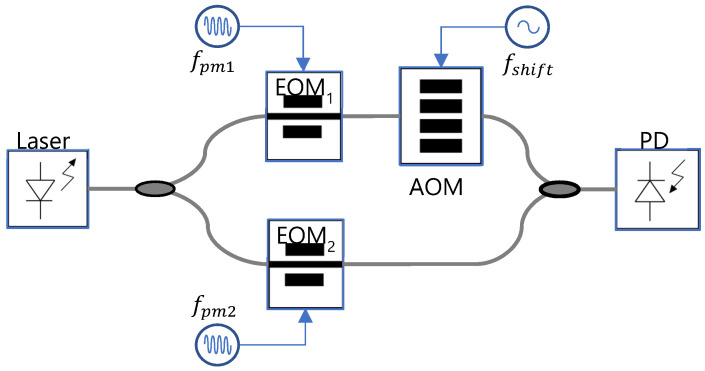
Basic scheme of an electro-optic dual optical frequency comb connected to a photodetector. EOM, electro-optic phase modulator; signal generators of EOM at frequencies *f_pm_*_1_ and *f_pm_*_2_; AOM, acousto-optic modulator; signal generator of AOM at frequency *f_shift_*; PD, photodetector.

The schematic of Figure 1 shows the architecture of an electro-optic DOFC connected to a photodetector. The laser seed of frequency *f*_0_ splits into two arms, each one corresponding to an optical frequency comb generated by an electro-optic phase modulator (EOM). The frequency applied to EOM_1_ is slightly different to the frequency applied to EOM_2_. The optical frequency of one arm is shifted by an acousto-optic modulator (AOM). Both arms are combined to beat the two optical frequency combs and, as a result, a multi-heterodyne interferometer is obtained. The signal revealed on a photo-detector is a replica of the optical frequency comb around $f_{0}$ (frequency spacing: $f_{pm1}$ − $f_{pm2}$) that is downshifted to a frequency comb around $f_{shift}$ (frequency spacing: $f_{pm1}$ − $f_{pm2}$). Note that $f_{0}$ >> $f_{pm1}$, $f_{pm2}$, $f_{shift}$ and that the two combs are coherent because they come from the same highly coherent seed.

**Figure 2:**
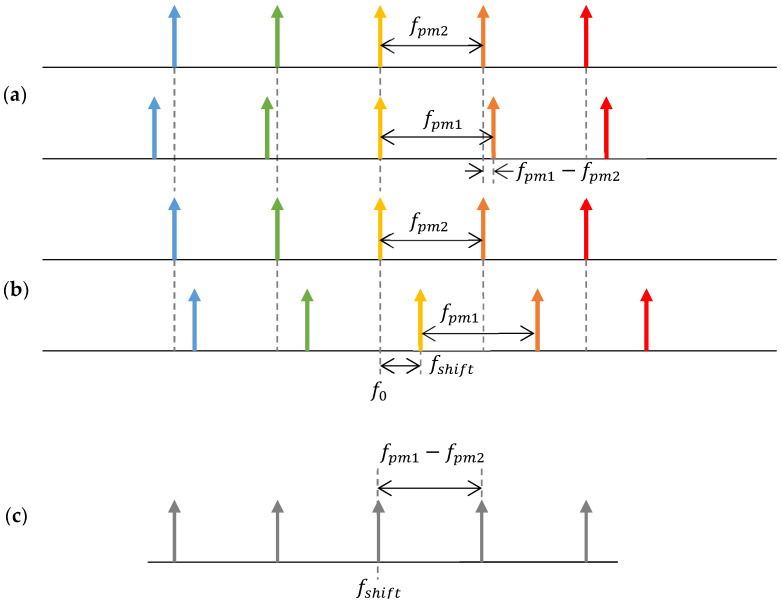
Electro-optic dual optical frequency comb spectra: (**a**) two optical frequency combs of slightly different frequencies applied to the phase modulator; (**b**) two optical frequency combs with additional frequency shifts; and (**c**) photo-detected comb as the beat response of the dual comb of (**b**).

The DOFC generates a multimode multiheterodyne optical signal that has an injective mapping from the optical spectrum probe (THz frequency, μm wavelength) to the electrical spectrum analyzer within a moderate bandwidth of a photodetector (kHz—MHz). Figure 2 shows the principle of an electro-optic DOFC generation and detection. Figure 2a shows the non-shifted optical frequency combs generated by two EOM to illustrate the frequency difference among each generated tone. Figure 2b shows the AOM shifted the optical frequency combs that allow an unambiguous beating of each tone pair on a unique frequency. Note that $f_{pm1}$ − $f_{pm2}$ << $f_{pm1}$,$\;f_{pm2}$ and $f_{shift}$ << $f_{pm1}$,$\;f_{pm2}$. Finally, the DOFC read by a photo-detector is shown in Figure 2c. The tone of frequency $f_{shift}$ corresponds to the optical central frequency $f_{0}$ (wavelength $\lambda_{0}=c/f_{0}$, c is the speed of light in vacuum), and the $f_{pm1}-f_{pm2}$ frequency spacing corresponds to the $f_{pm2}$ optical frequency spacing (wavelength spacing:$\Delta \lambda ={\left(\lambda_{0}\right)}^{2}\cdot f_{pm2}/c\;\;\;{\left(\lambda_{0}\right)}^{2}\cdot f_{pm1}/c$).

### 2.2. Compact Dual-Drive Electro-Optic Dual Optical Frequency Comb

**Figure 3:**
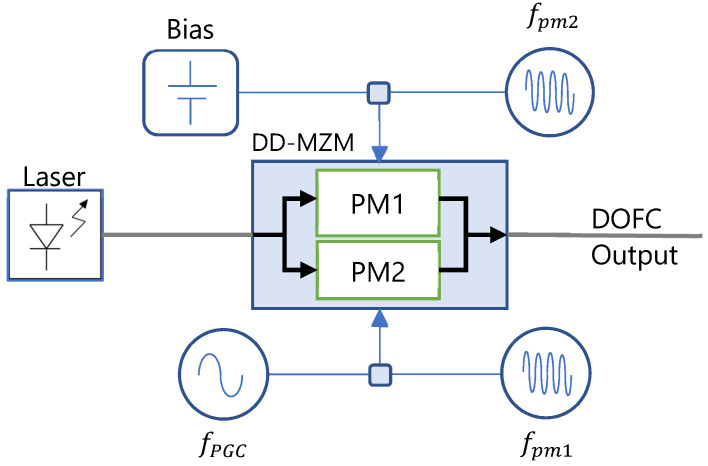
Basic scheme of a dual optical frequency comb (DOFC) generated by a dual-drive Mach–Zehnder modulator (DD-MZM) and phase-generated carrier (PGC). PM, phase modulator.

The generation of the DOFC is itself a worthy paradigm and state-of-the-art research is struggling to improve the characteristics of the resulting spectra in terms of bandwidth, flatness, coherence and stability. The electro-optic DOFC of Figure 1 is portable and has been implemented in practical applications. However, this set-up is a fiber interferometer where the stability of the generated optical combs depends on the fiber arms. Instead, we propose to use a dual-drive Mach–Zehnder modulator (DD-MZM) with the modulation scheme shown in Figure 3 to obtain a DOFC.

We have also three signals to generate the DOFC. Two of them are provided by two signal generators at frequencies $f_{pm1}\;$ for the first comb and $f_{pm2}\;$ for the second comb as in Figure 2a. In this case, the frequency shift is obtained through a phase-generated carrier (PGC) by applying an additional generator at frequency $f_{PGC}$ to one input of the DD-MZM. In addition, a bias input that can adjust the steady-state point of operation of the interferometer is available.

As before, frequencies $f_{pm1}$ and $f_{pm2}$ are slightly different and much higher than frequency $f_{PGC}$. If the signal generators of $f_{pm1}$ and $f_{pm2}$ are switched off, the scheme is essentially a pseudo-heterodyne interferometer with a phase-generated carrier [16]. The bias input adds another degree of freedom. It is used for choosing the phase difference between the first and second arms of the interferometer. This parameter, in addition to the set-up compactness, is important to improve the amplitude stability of the generated optical frequency combs and the interference between them.

The PGC technique in interferometry is based on generating an electrical carrier through phase modulation [21]. It can be also achieved by modulating the wavelength of the laser to generate a pseudo-heterodyne signal for a given optical path difference [22]. In our case, we used an additional phase modulation sine signal on one input of the DD-MZM. This non-linear process generates several carriers [22] and each one provides a pseudo-heterodyne detection. The resultant DOFC frequency shift is equivalent to those provided by the AOM. The particularity, in this case, is that, instead of a frequency comb centered on $f_{shift}$ (frequency spacing: $f_{pm1}$ − $f_{pm2}$), we obtained, on the photo-detector, a set of frequency combs: the first and principal one is centered on $f_{PGC}$ (frequency spacing: $f_{pm1}$ − $f_{pm2}$) and the others on 2·$f_{PGC}$ and 3·$f_{PGC}$. Note that each of these combs have the same frequency spacing of $f_{pm1}$ − $f_{pm2}$ and are an injective mapping of the DOFC.

The underlying concept in this process is the three pure-phase modulation stages performed in an interferometer. From the basic interferogram equation [23], we can consider three pure-phase modulations, two for the sidebands of the optical combs and one for the PGC approach. Although this is simple, this approach enables us to understand the process of sideband generation and, at the same time, the PGC process [24,25,26,27] as a simple Equation (3). It provides an expression for the detected intensity of the DOFC based on the PGC.

(3) $$I_{DOFC}=\|A_{1}\|{}^{2}+\|A_{2}\|{}^{2}+2\|A_{1}\|\|A_{2}\|\cos\left(\beta_{3}\sin\left(\omega_{3}t\right)+\beta_{1}\sin\left(\omega_{1}t\right)-\beta_{2}\sin(\omega_{2}t\right)+\phi_{1}\left(x\right)-\phi_{2}\left(x\right))$$

where $\|A_{1}\|{}^{2}$ is the power of one of the DD-MZM arms and $\|A_{2}\|{}^{2}$ is the power of the second arm of the DD-MZM. $\beta_{i}$ is the modulation depth of the *i*-th modulation and $\omega_{i}$ is the angular frequency of the *i*-th modulation. For convenience, the $\beta_{3}\sin\left(\omega_{3}t\right)$ term is associated with a pure-phase-modulation phase-generating carrier signal whose angular frequency $\omega_{3}$ is very small in comparison to the sideband-generating angular frequencies $\omega_{2}$ and $\omega_{1}$. As an example, in our practical case, 656.5 MHz and 656 MHz for $\omega_{1}/2\pi$ and $\omega_{2}/2\pi$, respectively, and 4 MHz for $\omega_{3}/2\pi$. $\phi_{1}\left(x\right)-\phi_{2}\left(x\right)$ represent the phase difference between the arms of the DD-MZM, and it is physically controlled by the bias signal.

The Fourier decomposition of (4) provides a useful insight. It enables us to understand the relationship between the optical combs and the photo-detected comb.

(4) $$I_{DOFC}\propto 2\|A_{1}\|\|A_{2}\|\overset{\infty }{\underset{m=-\infty }{\sum }}\left\{\overset{\infty }{\underset{\;k=-\infty \;}{\sum }}J_{m}\left(\beta_{3}\right)J_{k}\left(\beta_{2}\right)J_{k}\left(\beta_{1}\right)\cos\left[k\left(\omega_{1}-\omega_{2}\right)t+m(\omega_{3}t\right)+\phi_{1}\left(x\right)-\phi_{2}\left(x\right)]\right\}$$

where the “*m*-th” index sum is associated with the PGC-carrier generation of modulation depth $\beta_{3}$ and angular frequency $\omega_{3}$. Accordingly, the “*k*-th” index is associated to each homolog pair of tones that are mapped to the detector bandwidth. $k\omega_{1}$ and $k\omega_{2}$ harmonics are assumed to lie outside the detected bandwidth, and therefore they can be neglected. Therefore, just the carriers corresponding to $\omega_{3}$ and their multiheterodyne sidebands remain detectable. Otherwise, the residual harmonics can be easily removed with low-pass filtering. Each $\|A_{1}\|\|A_{2}\|J_{m}\left(\beta_{3}\right)J_{k}\left(\beta_{2}\right)J_{k}\left(\beta_{1}\right)$ term refers to the optical power for the “*k*-th” homolog tones measured in the “*m*-th” PGC carrier. Therefore, the relationship between the electrical domain ($I_{DOFC}$) FFT and the optical domain is injective (one to one mapping).

### 2.3. Multiheterodyne Dispersion Interferometer

**Figure 4:**
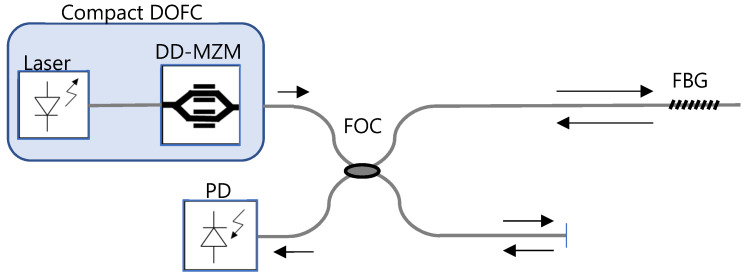
Basic scheme of the multiheterodyne dispersion interferometer for interrogating a fiber Bragg grating (FBG). DOFC, dual optical frequency comb; DD-MZM, dual-drive Mach–Zehnder modulator; FOC, fiber optic coupler/splitter; PD, photo-detector. The arrows indicate the direction of propagation of the light.

The scheme of the proposed multiheterodyne dispersion interferometer is shown in Figure 4. A compact DOFC based on a coherent laser seed and a DD-MZM (as in Figure 3) is injected into a Michelson interferometer with an FBG sensor in one arm. The central wavelength of the optical comb is aligned with the Bragg wavelength of the FBG sensor.

The light mix of the interferometer is composed of a variable amplitude and phase-signal correspondent with the reflection from the FBG sensor and a constant amplitude and phase-signal correspondent to the reference arm of the Michelson interferometer that is partially reflective.

The optical phase difference of the photo-detector depends on the source frequency:

(5) ϕ(ν)=2π·d·νc;       ϕ(λ)=2π·dλ

where ϕ is the optical phase difference between the two paths of the interferometer, *d* is the optical path difference (OPD) that considers both the length and the refractive index of the fiber, ν is the optical frequency, *c* is the speed of light in vacuum and λ is the optical wavelength.

In this case, each tone of the optical comb is detected with a different optical phase, and the phase difference of each tone with the central tone of frequency $f_{0}$ (wavelength $\lambda_{0}$) can be expressed as in (6).

(6)  ϕ(λ)−ϕ0=2π·(λ−λ0)·dλ02=2π·dc·(f−f0)

where $\phi_{0}$ is the optical phase difference at frequency $f_{0}$ (wavelength $\lambda_{0}$) that corresponds to the laser wavelength and $\left(\lambda -\lambda_{0}\right)$ << $\lambda_{0}$. Note that if the OPD is zero, then the optical phase of each and every tone is the same. The optical phase difference between the adjacent tones is 2π·dc·fpm, where $f_{pm}$ is the modulation frequency applied to the phase modulators (see Figure 2).

**Figure 5:**
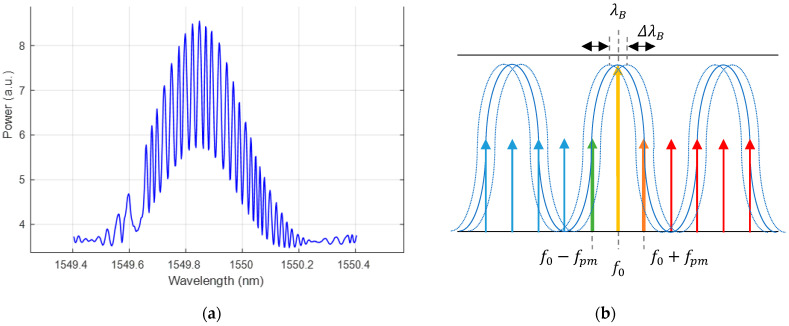
Principle of measurement based on a multiheterodyne dispersion interferometer to interrogate an FBG: (**a**) example of photo-detected output of a dispersion interferometer with an FBG obtained with a SLED and an optical spectrum analyzer (OSA); (**b**) sampling of specific wavelengths with a DOFC for an optical phase read-out.

In Figure 5a, we reproduce the output of the interferometer substituting the pair DOFC and PD by a low-coherent broadband source and an optical spectrum analyzer (OSA) as an example of the interferometer output as a function of the wavelength [28]. The interferometer output contains the amplitude profile of the FBG reflected spectrum and also the sinusoidal modulation with the wavelength change. When the reflected Bragg wavelength changes with the strain, the sinusoidal modulation shifts. Therefore, to obtain the strain, we can sample specific wavelengths that are representatives for an optical phase read-out, as it is represented in Figure 5b. In particular, the case of using the two tones $f_{0}+f_{pm}$ and $f_{0}-f_{pm}$ with the π rad optical phase difference between them and the DOFC central wavelength is aligned with the FBG Bragg wavelength.

In Figure 5a, an asymmetric spectral structure is observed. The representation is a combination of the envelope presented by the filtered/reflected spectrum of the FBG and the phase change with the wavelength of the FBG (i.e., delay) detected with a dispersion interferometer. The delay in this FBG is constant with the wavelength (minimum dispersion), but an excess is typical on the passband sides of an FBG without apodization [11]. In this case, zero path imbalance is obtained for a wavelength other than the Bragg wavelength, and the phase-wavelength profile is shifted from the amplitude-wavelength profile. Furthermore, the shorter and longer wavelengths do not reflect at exactly the same point on the FBG, so the path imbalance slightly changes for different wavelengths.

## 3. Methods

### 3.1. Experimental Set-Up

The experimental set-up reproduces the scheme of Figure 4 for the interrogation of a weak FBG with a multiheterodyne dispersion interferometer. The DOFC reproduces the scheme of Figure 3 for a compact and stable implementation. The system is driven with a laser 1310 nm wavelength (Santec Tsl-210 tunable laser). The DOFC is generated with a DD-MZM (model MZDD-LN-10-PD-P-P-FA-FA, iXblue, Saint Germain-en-Laye, France); an input is driven by a signal of 656 MHz, the other by both a signal of 656.5 MHz and a signal of 4 MHz, the latter for the PGC.

**Figure 6:**
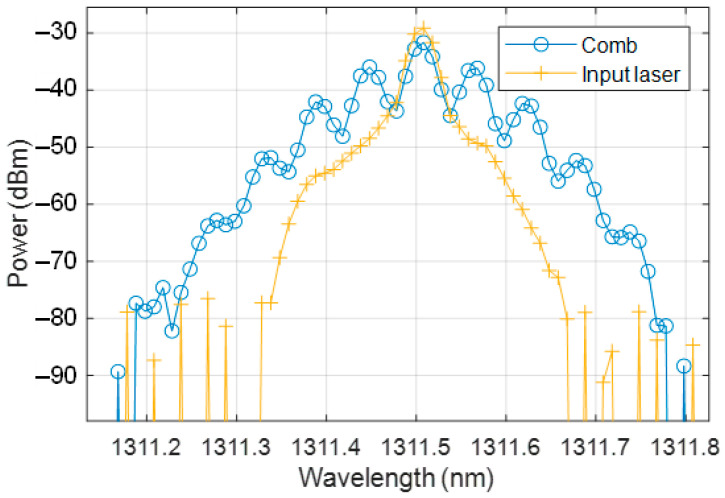
Generated dual optical frequency comb detected in an OSA.

An example of the optical output of the DOFC is shown in Figure 6, detected with an OSA (model Yokogawa AQ6370B). In this case, the modulation frequencies applied to the DD-MZM are higher in order to distinguish the different tones with the limited resolution of the OSA (20 pm resolution). The wavelength of the laser seed was about 1311.5 nm and the power was greater than −30 dBm. The central wavelength of the comb was the same (1311.5 nm) and the power was slightly less than −30 dBm. The amplitude of the harmonics was larger in the comb trace compared to the laser trace, since the EO modulator generated sidebands, but the total comb power was less than the laser power due to insertion losses.

The fiber was SMF-28. The fiber-optic coupler was a 50:50 dual-wavelength (1310 nm, 1550 nm). The FBG sensor was a weak FBG of 2 cm, low back reflectivity of 12.5% and 1308.2 nm reference Bragg wavelength.

The central wavelength of the optical comb was aligned with the Bragg wavelength of the FBG sensor by adjusting the wavelength of the laser. During the experiments, it was adjusted before the measurements to obtain an identical amplitude of the first sidebands. In practice, the effect of the thermal drift of the Bragg wavelength can be compensated through the tunability of the laser by implementing a low-bandwidth closed loop. Furthermore, if 5 optical-phase sample points (5 comb lines) are obtained [28], the optical phase change can be reconstructed over a range of more than 2π rad, so vibrations and drift can be measured simultaneously to compensate for the latter.

The tones of the comb were spaced at 656 MHz to have a phase difference of π/2 rad between the two adjacent tones, so the optical path difference of the fiber interferometer was 11.43 cm. Since the light traveled along the fiber forward and backward (two times) and considering an effective refractive index of 1.4676 at 1310 nm, the length difference between the reference fiber and the FBG reflection was 3.90 cm.

The frequency difference between the first and second comb was imposed to be 0.5 MHz, which was the limit for the FBG sensor’s detected bandwidth without interference among the tones in the photodetector. This frequency difference was far less than the modulation frequency of 656 MHz, so both optical combs sampled the same point of the optical spectrum (the phase difference was approximately 381 ppm of π rad).

The PGC was modulated at 4 MHz and the amplitude was chosen to have 2 Vπ of the phase modulator, which was approximately 7 V. This means that, for an amplitude equal to Vπ = 3.5 V, we obtained the π radians of the optical phase modulation. Therefore, for 2 Vπ, we obtained a whole period of modulation for each period of the phase modulation. In this case, with a frequency shift of 4 MHz and a frequency difference of 0.5 MHz, up to 8 tones can be read unambiguously on the photodetector. As can be observed in Figure 6, the DOFC has 7 tones within 30 dB of the relative amplitude and the other tones are negligible.

The photo-detecting stage is a self-made bank of photodetectors with 35 dB of gain that can operate at 1310–1550 nm wavelengths.

We chose the wavelength of 1310 nm of the tunable laser and FBG samples; other wavelengths, such as 1550 nm, can be used with this configuration [28]. An advantage is the reduced dispersion of the fiber cables at 1310 nm, which implies that a model in which the optical phase is constant with the wavelength is representative. Regarding the comb line spacing, 656 MHz was chosen to accurately place the two main sidebands $f_{0}$+$f_{pm}$ and $f_{0}$ − $f_{pm}$ in a specific optical phase of ±π/2 rad, with respect to the reference (Figure 5b). Furthermore, this frequency was moderate, so it satisfied $f_{shift}$<< $f_{pm1}$, $f_{pm2}$ (4 MHz << 656 MHz, 656.5 MHz) and $f_{pm1}$ − $f_{pm2}$<<$f_{pm1}$, $f_{pm2}$ (0.5 MHz << 656 MHz, 656.5 MHz).

**Figure 7:**
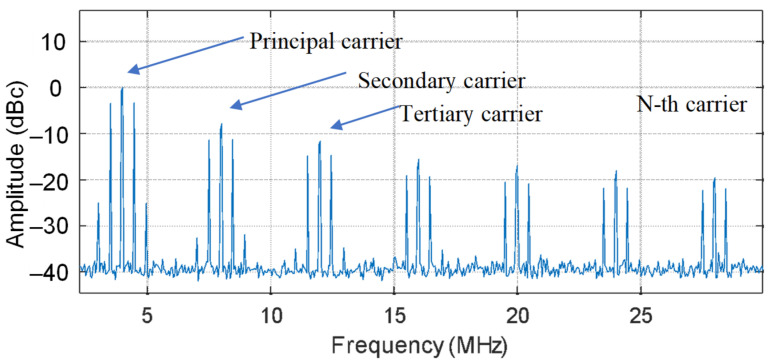
DOFC read-out on the photo-detector. Detected signal with multiple read-out combs, each one centered on one carrier of the multiple carriers generated by the PGC.

As previously mentioned, multiple carriers were generated with the PGC, each one mapping the optical comb to a comb on the photodetector. Therefore, the optical comb was mapped as a comb with a frequency spacing of 0.5 MHz centered on 4 MHz and equivalent combs (0.5 MHz spacing frequency) centered on the harmonics of 4 MHz (such as 8 MHz and 12 MHz). This injective mapping from the DOFC to the photodetector signal can be observed in Figure 7. The PCG characteristic of the modulation lead to multiple carriers and, therefore, the same spectra are injective and mapped along the spans of 2–6 MHz, 6–10 MHz, 10–14 MHz and 14–18 MHz for the first order, second order, third order and fourth order, respectively. In this case, 5 tones of the optical comb were clearly detected in the principal and secondary carriers of 4 MHz and 8 MHz, respectively, and 3 tones of the optical comb were detected in higher-order harmonics of 4 MHz.

### 3.2. Demodulation

The demodulating process can be explained over one wavelength, and generalized to each comb tone on the photodetector (PD). The vibration tone resulting from the wavelength $\lambda_{i}$ can be extracted from the PD by mixing the signal of PD (SPD) with the reference mixing signal (SR) and filtering the output with a low-pass filter. In this case, we used an analog mixer whose output was proportional to the SPD times of the mixer reference signal SR. If the SR had a constant amplitude and the same frequency as the understudy SPD harmonic, we obtained an electrical output amplitude that was proportional to the amplitude modulation of the optical tone lying in the FBG reflected spectrum. Thus, the fluctuation of this amplitude will be at the same rate as the frequency of the mechanical vibration.

The vibration information was extracted from the principal carrier with the differential measurements of the amplitudes of the harmonics at 3.5 MHz and 4.5 MHz. By applying the lock-in technique to those two tones and subtracting them, we obtained a value of the phase shift of the interferogram. This simplification is extremely important in the dynamic-strain measurement with the FBG.

**Figure 8:**
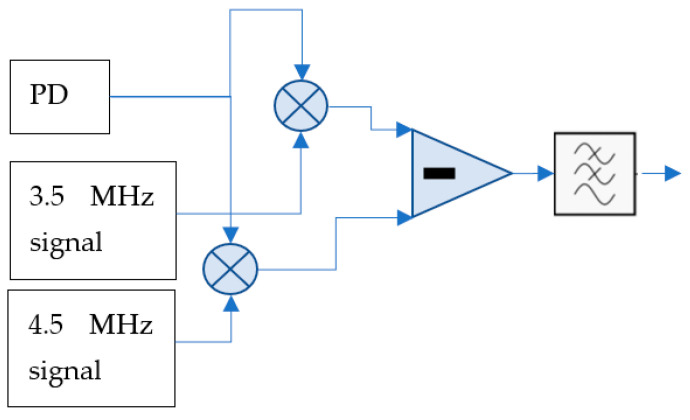
Demodulation algorithm implementation with analog mixers, differential stage and low-pass filtering. PD, photodetector at the output of Figure 4.

In Figure 8, we can observe the implementation of the analog demodulation stage.

The PD signal is split and injected into analog mixers that are driven with the other secondary signals of 3.5 MHz and 4.5 MHz, respectively. The output of each mixer contains the beat in DC and twice the frequency. Thus, at the output of the low-pass filter, we obtain the electrically encoded vibration signal whose amplitude is proportional to the mechanical vibration signal around the DC.

### 3.3. Calibration

The calibration system is performed with an independent heterodyne interferometer. Both sensing parts were mechanically attached to the same point and in the same way to ensure that the same strain and vibration stimuli was applied to both the calibration interferometer and the main measurement system. Each system was driven with a different laser: the main measurement system was driven with a tunable laser (Santec Tsl-210) and the calibration system was driven with a laser diode (QDFB-LD-1550-20).

**Figure 9:**
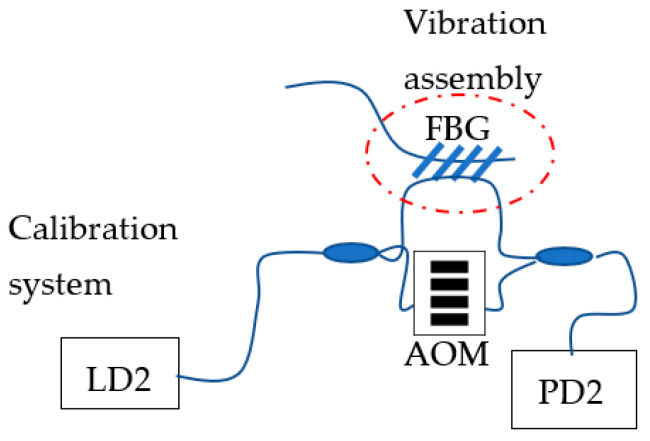
Vibration assembly for the calibration system and FBG sensor. LD2, laser diode of the calibration interferometer; PD2, photodetector; AOM, acousto-optic modulator.

A heterodyne interferometer purely modulated in the phase on one of its arms generated electrical sidebands around the carrier signal. Their spacing of the sidebands was the same as the sinusoidal excitation frequency. As the measurement arm of the calibrating interferometer and the FBG sensor were mounted in the same assembly, both parts experienced the same mechanical displacement (Figure 9). The number and amplitude of the sidebands generated in the interferogram of the calibrating interferometer depends on the amplitude of the mechanical vibration signal.

We adjusted the mechanical set-up to ensure that the FBG sensor and the total sensing path of the calibrating interferometer were the same, so we could easily obtain an absolute amplitude for the vibration mechanical system that was independent of the main system. This allowed us to measure the minimum resolution of the main system from the output of the calibration system in PD2.

The normalized optical fiber path length change with the strain (7) was in the range of the relative Bragg wavelength change of the FBG.

(7) ΔnLnL=KF·ε=KF·ΔlL0,

where *nL* is the optical path length of the sensing piece of fiber, *n* is the effective refractive index, Δ*nL* is the change of this path length and $K_{F}$ is the gage factor of the optical fiber that considers the strain-optic coefficient. Considering a practical gage factor of 0.78 and a 1.4682 refractive index for the 1550 nm wavelength in the SMF-28 silica fiber, the optical path elongation was 1.16 times that of the physical elongation of the fiber. A change of Δ*nL* equal to the laser wavelength produced a 2π rad optical phase change.

The calibration process was very simple and allowed us to measure the resolution limits of the system for the dynamic strain. The algorithm was explained in [29,30]. The idea is to measure the sideband attenuation between the zero and first-order Bessel functions of the heterodyne interferometer. This attenuation is proportional to the modulation depth and therefore to the ratio between the wavelength of the laser diode (LD2) and the actual amplitude of the mechanical vibration.

**Figure 10:**
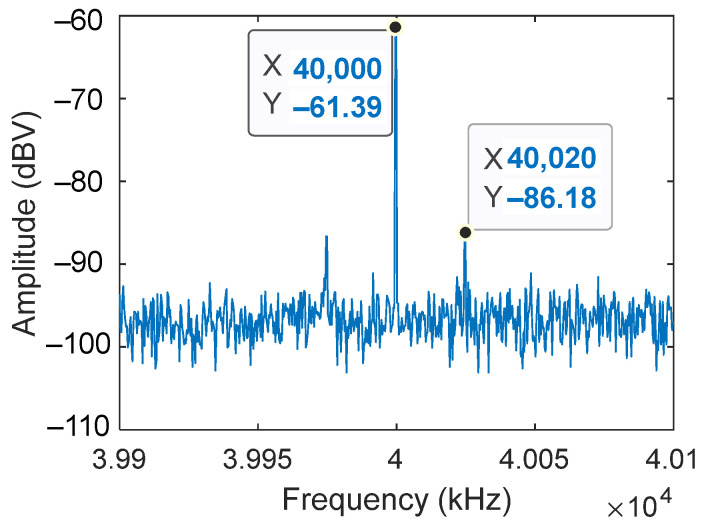
Reference of the calibration obtained with the heterodyne interferometer for an excitation of the PZT transducer of 20 V and 20 kHz. Signal on the photodetector PD2.

To obtain a calibration level that shows a value of applied strain, we applied a known value of amplitude to the PZT. The resulting strain generated sidebands over the calibrating signal that was easily transformed into the absolute strain. A particular strain value is shown in Figure 10 as an output response of the calibrating system for 20 V applied at a 20 kHz frequency.

A ratio of about 25 dB between the zero and the first harmonic was achieved. It led to 0.1 rad over a 10 cm length of fiber that was 215 nε, measured with the heterodyne interferometer at 20 kHz and 20 V of amplitude.

## 4. Results

The set-up compactness by a single dual-drive-unit optical modulator is important to improve the stability of the optical frequency combs generated and the interference between them. The mapping quality is better in the case of dual-drive implementation than in a discrete component arrangement. This is because the impact of the temperature instabilities that arise from interferometric implementations are reduced to their minimum as the DD-MZM is intrinsically more stable than a fiber implementation. Therefore, less unwanted fluctuations of amplitude RF mapping were achieved.

**Figure 11:**
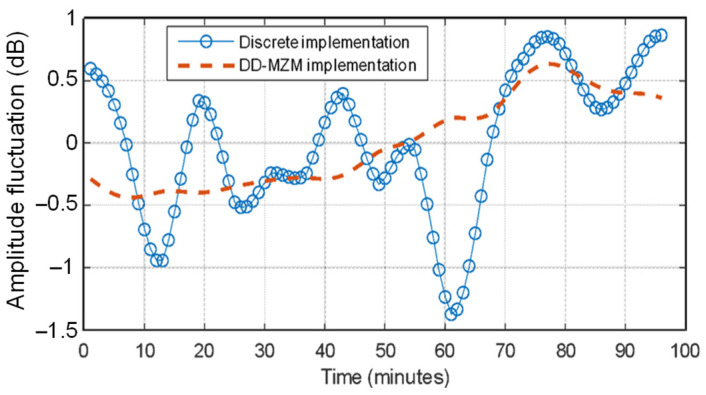
Amplitude stability of the proposed system with the EOM-AOM DOFC architecture and the DD-MZM in-chip DOFC architecture.

To support this conclusion, we analyzed the amplitude stability during a total period of 100 min. We registered the amplitude of two free-running DOFC: an EOM-AOM implementation, following the scheme of Figure 1, and a DD-MZM implementation, following the scheme of Figure 3. The measurements of both systems were made at the same time. We used controlled laboratory conditions on the same table under a similar environment. The results of both DOFC can be seen in Figure 11.

From this, we can extract that the maximum fluctuation of the discrete implementation is bigger than the same DD-MZM based implementation. In the case of the DD-MZM, the maximum value is about 0.5 dB, while in the case of discrete implementation, it is almost 1.5 dB. This implies that better amplitude stability on the optical source leads to a higher quality in the measurement of the output signal.

**Figure 12:**
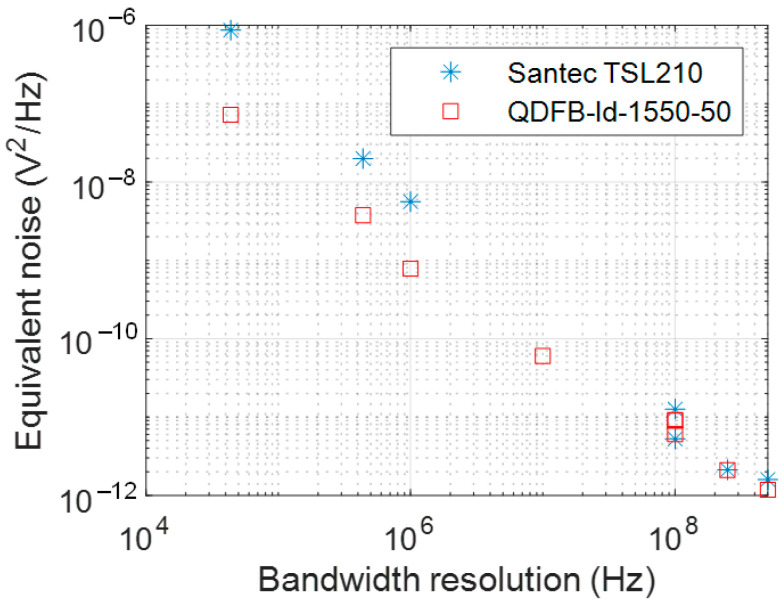
Equivalent noise as a function of the bandwidth resolution for both lasers.

On the other hand, we measured the noise levels for different bandwidth resolutions in order to determine the quality of the under study signal. We can observe in Figure 12 that, for a higher sampling rate (*x*-axis), we obtained a lower noise that was able to reach the levels of $20{\;\mathrm{pV}}^{2}/\mathrm{Hz}$, in the case of the Santec laser, and of about $10{\;\mathrm{pV}}^{2}/\mathrm{Hz}$, in the case of QDFB-LD-1550-50.

Finally, we applied small-amplitude high-frequency signals. Therefore, a small signal approach was used for very fast mechanical excitation and a linear amplitude modulation of the optical comb tones by the FBG sensor was assumed.

**Figure 13:**
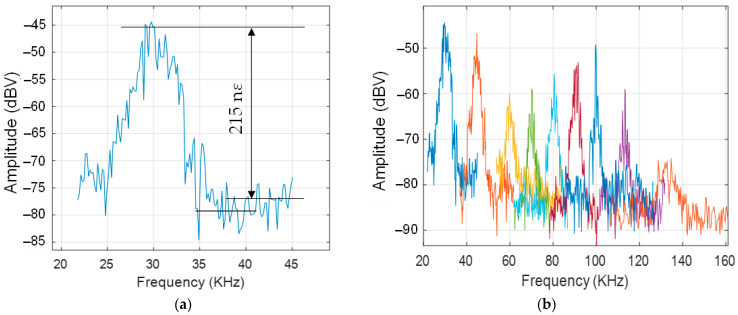
Detected vibration signals after the lock-in amplification stage: (**a**) calibrated measurement of the FBG dynamic strain at 30 kHz equivalent to 0.1 rad over a 10 cm length of fiber; (**b**) frequency sweep of the mechanical vibration.

Figure 13a shows the calibrated measurement at 30 kHz (ESA resolution bandwidth of 300 Hz). A dynamic strain in the range of 215 nε was detected with a SNR of 30 dB. The vibration signal was measured for several cases of different mechanical excitation frequencies to see the maximum operating bandwidth as shown in Figure 13b. That is, in our case, at about 130 kHz, it was defined by the maximum frequency of vibrations that are detectable with an SNR of 10 dB. The *y*-axis represents the electrical amplitude and the x-axis represents the frequency of the mechanical excitation. Each color represents a different mechanical excitation frequency.

It can be observed in Figure 13a that the bandwidth of the detected mechanical vibrations is widened with respect to the excitation frequency. As can be seen in Figure 13b, the bandwidth of the detected vibrations is similar for different frequencies, so the same amplitude/phase modulation of the main mechanical frequency is observed in all cases, which represents a common fluctuation. The phase noise of the laser seed (5 MHz bandwidth, FWHM) and the amplitude jitter of the reference comb can be considered the sources of error. However, a continuous wave, rather than a burst, was used to excite the acoustic actuator, which could have contributed to this effect.

## 5. Conclusions

In this work, a dispersion reading system to interrogate low reflectivity FBG sensors and measure dynamic strain with a high resolution was presented. It was based on dual optical frequency comb generation (DOFC) and allowed a compact setup that operated with increased amplitude stability compared to classical discrete architectures. The DOFC was generated with a single device, a dual-drive Mach Zehnder modulator (DD-MZM), and provided a compact and usable alternative to the discrete architecture implementation. We used sideband generation and the phase generating carrier technique for reading dispersion variations due to sinusoidal vibration in a low reflectivity FBG sensor.

We detected a dynamic strain with amplitudes in the range of 215 nε and with a signal to noise ratio of 30 dB that were calibrated independently with a heterodyne interferometer. The main system also reached a maximum detectable frequency of 130 kHz with a signal to noise ratio of almost 10 dB.

As future work, we can point to the application of the dispersion reading to distributed grating measurements, that is, the measurement of the intra-grating strain as shown in [14,31,32] and intra-grating temperature [33]. These ideas rely on the fact that the dispersion may be dependent on the intra-grating position of the FBG sensor and, therefore, information about the position can be extracted for a distributed sensing of the strain. This information about the position is proportional to the first derivative of the phase with respect to wavelength, which is a magnitude that can be extracted with our proposed technique.

In addition, the narrow-band detection of the fiber sensor dispersion changes will enable the compact, cost-effective and high-resolution interrogation of high-throughput interferometric fiber sensors as integrated Match-Zehnder interferometer (MZI) sensors and long-period grating (LPG) sensors. These open also its application to the precise measurement of chemical/biological sensing samples at high speeds.

## Data Availability

The datasets of the current study are available from the corresponding author upon reasonable request.
